# Effect of therapeutic plasma exchange on endothelial activation and coagulation-related parameters in septic shock

**DOI:** 10.1186/s13054-020-2799-5

**Published:** 2020-03-02

**Authors:** Klaus Stahl, Julius J. Schmidt, Benjamin Seeliger, Bernhard M. W. Schmidt, Tobias Welte, Hermann Haller, Marius M. Hoeper, Ulrich Budde, Christian Bode, Sascha David

**Affiliations:** 1 Department of Gastroenterology, Hepatology and Endocrinology, Hannover Medical School, Hannover, Germany; 2 Department of Nephrology and Hypertension, Hannover Medical School, Carl-Neuberg-Str.1, 30625 Hannover, Germany; 3 Department of Respiratory Medicine and German Centre of Lung Research (DZL), Hannover Medical School, Hannover, Germany; 4 Medilys Laborgesellschaft mbH, Hamburg, Germany; 5 Department of Anaesthesiology and Critical Care, University Medicine Bonn, Bonn, Germany

**Keywords:** Extracorporeal treatment, Septic shock, Plasmapheresis, ADAMTS-13, von Willebrand factor

## Abstract

**Background:**

A dysbalanced coagulation system is part of the pathological host response to infection in sepsis. Activation of pro-coagulant pathways and attenuation of anti-coagulant activity ultimately lead to microvascular stasis and consequent organ failure. No treatment approaches specifically targeting this axis are available. We explored the effects of therapeutic plasma exchange (TPE) on microvascular coagulation dysbalance in septic shock.

**Methods:**

We conducted a prospective single-center study enrolling 31 patients with early septic shock (onset < 12 h) requiring high doses of norepinephrine (NE > 0.4 μg/kg/min). Clinical and biochemical data, including measurement of protein C; a disintegrin and metalloprotease with a thrombospondin type 1 motif, member 13 (ADAMTS13); and von Willebrand factor antigen (vWF:Ag), were obtained before and after TPE against fresh frozen plasma.

**Results:**

Antithrombotic acting proteins such as antithrombin-III (ATIII) and protein C were markedly reduced in septic patients, but their activity increased after TPE (ATIII, 51% (41–61) vs. 63% (48–70), *p* = 0.029; protein C, 47% (38–60) vs. 62% (54–69), *p* = 0.029). Median ADAMTS13 activity was increased by TPE from 27 (21–42) % before to 47 (38–62) % after TPE (*p* < 0.001). In contrast, vWF:Ag was elevated and could be reduced by TPE (353 (206–492) IU/dL vs. 170 (117–232) IU/dL, *p* < 0.001). Regression analysis yielded a correlation between ADAMTS13 activity and platelet count (*p* = 0.001, *R*^2^ = 0.316).

**Conclusions:**

Septic shock was associated with activation of pro-coagulant pathways and simultaneous depletion of anti-coagulant factors. TPE partially attenuated this dysbalance by removing pro- and by replacing anti-coagulant factors.

**Trial registration:**

ClinicalTrials.gov, NCT03065751. Retrospectively registered on 28 February 2017.

## Background

Sepsis is defined as a life-threatening organ dysfunction caused by a dysregulated host response to infection; if hypotension is refractory to volume resuscitation and serum lactate is elevated, it is termed septic shock [1]. In the absence of a specific intervention other than anti-infective drugs, mortality associated with septic shock is still as high as 60% [2]. The overwhelming host response consisting of cytokine release, attraction of inflammatory cells, and global endothelial activation is the key driver of morbidity and mortality [3]. Endothelial dysfunction leads to systemic aggregation of platelets in virtually all microvascular beds with consecutive consumption of coagulation factors. Clinically, this situation is well known as disseminated intravascular coagulation (DIC) resulting in widespread clotting of the microvasculature system and progressive multi-organ failure [4].

The critical role of acquired protein C deficiency in septic shock with purpura fulminans has been recognized for a long time [5–8], and some experts suggest to supplement protein C if its activity is severely lowered [8]. Interestingly, the only ever approved “specific sepsis therapeuticum” was an activated protein C preparation (Xigris®) [9], before it was later removed from the market due to a large-scale negative follow-up study [10].

A disintegrin and metalloprotease with a thrombospondin type 1 motif, member 13 (ADAMTS13) is the most important modulator of size and function of the von Willebrand factor antigen (vWF:Ag) in human plasma by cleaving preformed vWF multimer chains [11]. Severe ADAMTS13 deficiency causes accumulation of ultra-large vWF multimers (ULVWF) leading to the clinical picture of severe thrombotic microangiopathy as seen in thrombotic thrombocytopenic purpura (TTP) [12]. More recently, it has been demonstrated that a significant deficiency of ADAMTS13 also appears in sepsis [13–15]. At the same time, large amounts of vWF:Ag are secreted by the activated septic endothelium leading to both increased platelet aggregation and formation of highly pro-thrombotic ULVWF [16]. In fact, an increased vWF:Ag/ADAMTS13 ratio has been repeatedly associated with the severity of shock and organ failure as well as increased mortality in sepsis [13, 17–20].

Our group has recently described the use of therapeutic plasma exchange (TPE) against fresh frozen plasma (FFP) as an adjunctive treatment strategy in early (onset < 12 h) and severe (norepinephrine (NE) dose > 0.4 μg/kg/min) septic shock [21]. Although uncontrolled, we observed rapid stabilization of hemodynamics, improvement of fluid balances, and reduction of key pro-inflammatory cytokines and permeability factors. We believe that these positive surrogate effects can be attributed to two important aspects: (1) removal of harmful circulating molecules and (2) replacement of protective plasma proteins consumed by the disease process [22].

Here, we hypothesize that TPE against plasma from healthy donors might (1) remove mediators of increased microvascular clotting (e.g., vWF with large and in many cases ultra-large vWF multimers) and (2) simultaneously compensate for the loss of factors important for regulating coagulation (e.g., activated protein C, ATIII) and fibrinolysis (e.g., ADAMTS13). In this study, we investigated in patients with early and severe septic shock the effect of a single TPE against FFPs on key factors regulating coagulation.

## Methods

### Study population

**Fig. 1:**
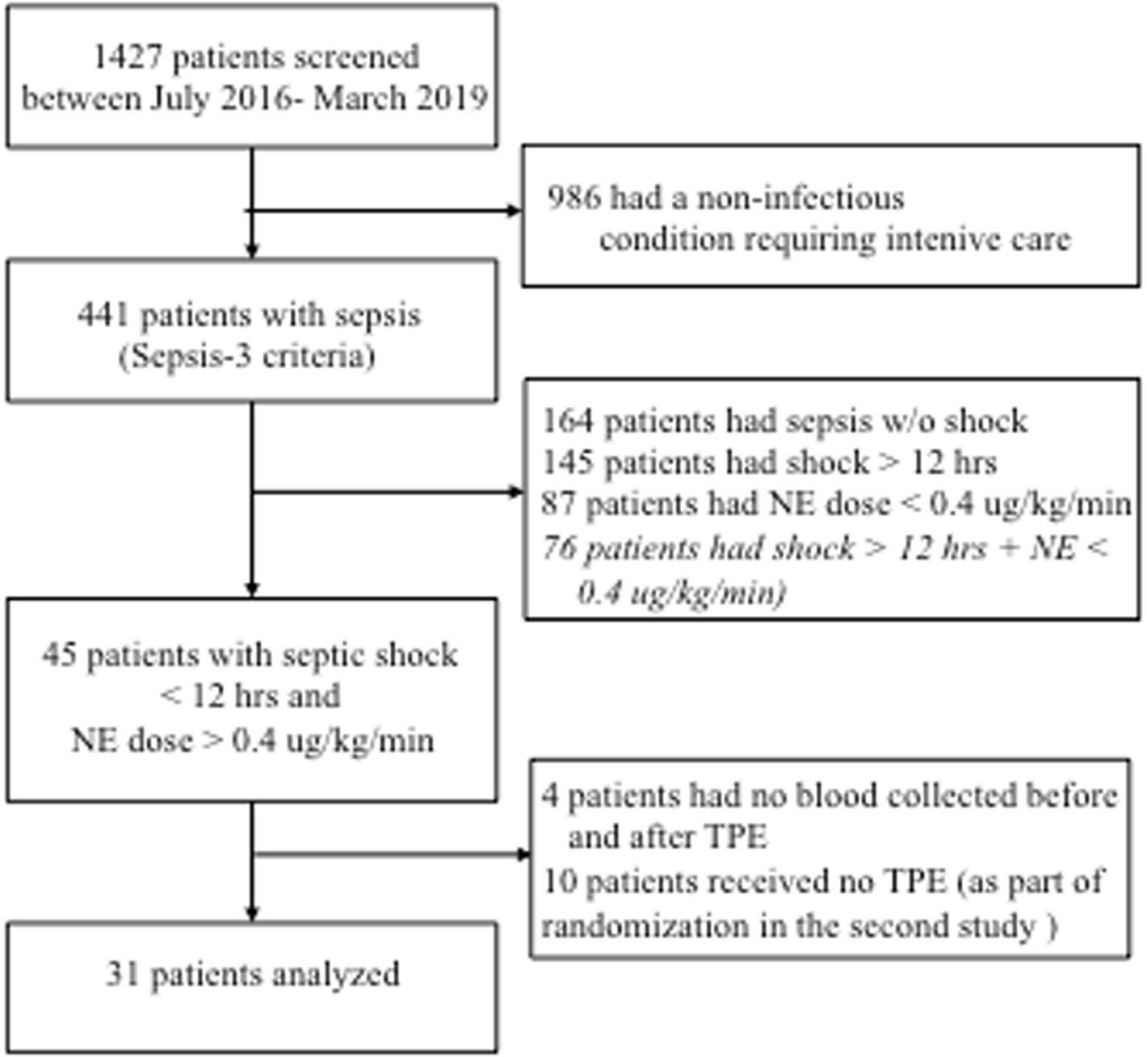
Flow chart of study participants. NE, norepinephrine; TPE, therapeutic plasma exchange

Data and bio-samples were acquired from both a recently completed prospective single-center non-randomized study [21] and a currently still-recruiting single-center randomized study (NCT03065751). We screened *n* = 1427 patients submitted to our 14-bed medical ICU from July 2016 to March 2019 for the presence of sepsis per SEPSIS-3 definition [1] (Fig. 1). Twenty patients from the published feasibility trial and 11 patients from the subsequent ongoing randomized trial were included. All patients were treated according to the 2016 Surviving Sepsis Campaign (SSC) guidelines. The ethical committee of Hannover Medical School approved both study protocols, and written informed consent was obtained from participants or authorized representatives. The study was performed in accordance with the ethical standards laid down in the 1964 Declaration of Helsinki and its later amendments. Demographic and clinical data were obtained immediately before TPE.

### Inclusion and exclusion criteria

Patients were included based on (i) septic shock with a need for vasopressors < 12 h prior to entry and (ii) profound systemic hypotension requiring norepinephrine (NE) doses of > 0.4 μg/kg/min despite adequate intravenous fluid resuscitation. All patients received a crystalloid fluid replacement with at least 30 mL/kg body weight before TPE treatment. Fluid status was routinely assessed following a routine in-house standard operating procedure applying bedside transthoracic echocardiography (TTE) and measurement of inferior vena cava (IVC) diameter and/or stroke volume variation by pulse contour cardiac output (PiCCO) catheter. The median volume administered before TPE treatment was 3225 (2274–4479) mL. As non-inclusion criteria, we defined unavailability of TPE within the first 6 h after study inclusion, pregnancy or breastfeeding, age < 18 years, end-stage chronic disease, and presence of a directive to withhold life-sustaining treatment.

### Therapeutic plasma exchange

Vascular access was established by venous insertion of an 11-French two-lumen hemodialysis catheter. We used one single TPE, as hemodynamic improvements were achieved only with the first TPE in a previous pilot study [23]. Centrifugal TPE was performed against a fixed dose of 12 units of FFP; therefore, total volumes exchanged varied between 1 and 1.5 times the plasma volume. Median blood flow was 60 (55–63) mL/min. Anti-coagulation during TPE was achieved by regional citrate infusion. Post-filter calcium concentration was checked 15 min after starting the treatment as regulated by a local protocol at our institution. The citrate flow rate was adjusted to target post-filter ionized calcium concentrations of 0.5 to 0.6 mmol/L. Additionally, systemic blood gas analysis was performed every 2 to 4 h to exclude electrolyte and acid-base dysbalances. Immediately before TPE treatment implementation, anti-allergic intravenous prophylaxis with ranitidine and clemastine was administered to all patients. In patients with acute kidney injury (AKI), hemodialysis was interrupted for the duration of TPE (110 (93–120) min). Blood samples were drawn before and after TPE. Patients were closely followed for the next 28 days, and survival was recorded. NE dose was titrated every 10–15 min to maintain a mean arterial pressure (MAP) above 65 mmHg.

### Measurement of coagulation factors, ADAMTS13, vWF:Ag, and ULVWF

Patient plasma and serum blood samples were acquired before and after TPE. For blood sampling, 10 mL monovettes containing 1 mL of 0.13 mol/L sodium citrate was used, and plasma was obtained by centrifugation divided into aliquots and stored at − 80° until assayed. ADAMTS13 activity (ADAMTS13) was determined with the Technoclone ADAMTS13 activity ELISA kit. vWF antigen (vWF:Ag) was measured by an enzyme-linked immunosorbent assay [24]. vWF multimers were separated by gel electrophoresis (60 V, 16 °C, 15 h) using 1.7% and 1.3% LGT agarose (Sigma-Aldrich, Seelze, Germany), detected by a vWF antibody conjugated with horseradish peroxidase (Dako, Hamburg, Germany) and visualized by an enhanced chemiluminescence (ECL detection kit; Amersham, Freiburg, Germany) [25]. The gels were recorded by a CCD camera and evaluated by the AphaView Q Software (Alpha Innotech).

Scores assessing coagulopathy were calculated, namely the sepsis-induced coagulopathy (SIC) and the International Society on Thrombosis and Haemostasis (ISTH) score for overt disseminated intravascular coagulation (DIC score). The SIC score was determined according to the suggested system by Iba et al. [26] with a score of ≥ 4 (out of max. 6) indicating sepsis-induced coagulopathy. The ISTH score was calculated as described previously [27], with a score of ≥ 5 indicating overt DIC, and for D-dimer concentration, the following cutoff values were used: 0–0.5 mg/L = 0 points, 0.5–2 mg/L = 2 points, and > 2 mg/L = 3 points [28].

Clinical parameters were correlated with ADAMTS13 activity at the time of inclusion before TPE treatment was begun. For this purpose, patients with less severely reduced ADAMTS13 activity, e.g., ≥ 30%, were compared to those with more profoundly reduced ADAMTS13 activity, e.g., < 30%. The cutoff of 30% ADAMTS13 activity has been used before by Brunkhorst and coworkers [14].

### Statistical analysis

Data were presented as median with interquartile range (IQR). Two-tailed *p* values of less than 0.05 were considered to indicate statistical significance. Paired *t* test or Wilcoxon test (as appropriate) was utilized in order to compare longitudinal values before (pre) and after (post) TPE. Unpaired *t* test and Mann-Whitney test (for not normally distributed variables) were employed to compare unpaired values. For correlation analysis (ADAMTS13 activity to platelet count), non-linear regression analysis was performed and *R*^2^ value was calculated accordingly. Survival data were analyzed by log-rank test and visualized by Kaplan-Meier curves. We used GraphPad Prism 7 (Graph Pad, La Jolla, CA, USA) and SPSS Statistics version 25 (SPSS Inc., Chicago, IL, USA) for data analysis and graph generation.

## Results

### Cohort characterization

Demographic and clinical details are summarized in Table 1. Seventy-one percent of the patients were men, and the median (IQR) age was 53 (35–59) years. The lungs and the abdomen were the most common sites of infection. A causative pathogen was identified in 68% of the cases. All patients were treated with a combination of broad-spectrum antibiotics. Gram-negative and Gram-positive pathogens were the most commonly identified, and in 13% of patients, more than one pathogen was detected.

**Table 1:** Demographic and clinical characteristics at baseline

Category	Median (IQR)/*n* (%)
Age, years	53 (35–59)
Sex, *n* (%)	
Male	22 (71)
Female	9 (29)
BMI, kg/m^2^	26 (20.7–31.6)
Sepsis onset, *n* (%)	
Ambulant	19 (61.3)
Hospital	12 (38.7)
Side of infection, *n* (%)	
Pulmo	17 (54.8)
Abdomen	7 (22.6)
Urogenital	1 (3.2)
Soft tissue	4 (12.9)
Endocarditis	1 (3.2)
More than one	1 (3.2)
Identified pathogen, *n* (%)	
Gram+	5 (16.1)
Gram−	9 (29)
Fungi	2 (6.4)
Viral	1 (3.2)
More than one	4 (12.9)
Non-identified	10 (32.3)
SOFA	18 (16–20)
Norepinephrine dose, μg/kg/min	0.801 (0.561–1.079)
CRP, mg/L	290 (159–327)
PCT, μg/L	26 (14–73)
Mechanical ventilation, *n* (%)	29 (93.5)
Oxygenation index (PaO_2_/FiO_2_)	127 (92–195)
Renal replacement therapy, *n* (%)	21 (67.7)

Given are the demographic and clinical characteristics at the time of study inclusion before TPE treatment. Values are presented as median (25 to 75% interquartile range) or if categorical as numbers and percentages

*BMI* body mass index, *SOFA* Sequential Organ Failure Assessment, *CRP* C-reactive protein, *PCT* procalcitonin

The median (IQR) SOFA score was 18 (16–20). Shock was profound with requiring about twice as high NE doses than required by the inclusion criteria. All patients displayed signs of severe hyper-inflammation as indicated by high levels of C-reactive protein (CRP) and procalcitonin (PCT). Ninety-four percent were mechanically ventilated and had an oxygenation index (PaO_2_/FiO_2_) of 127 (92–195). AKI with a need for renal replacement therapy (RRT) was present in 68% of the patients at the time of inclusion.

TPE was well tolerated, and no severe complications of TPE such as anaphylactoid reactions, citrate-induced hypocalcemia, catheter-related complications, and transfusion-related lung injury were observed.

### Effect of TPE on routine coagulation parameters, antithrombin-III, protein C, and D-dimers

While INR non-significantly decreased (1.6 (1.3–1.9) vs. 1.3 (1.2–1.7), *p* = 0.089), PTT remained unchanged following TPE (52 (40–76) s vs. 51 (38–60) s, *p* = 0.084).

**Fig. 2:**
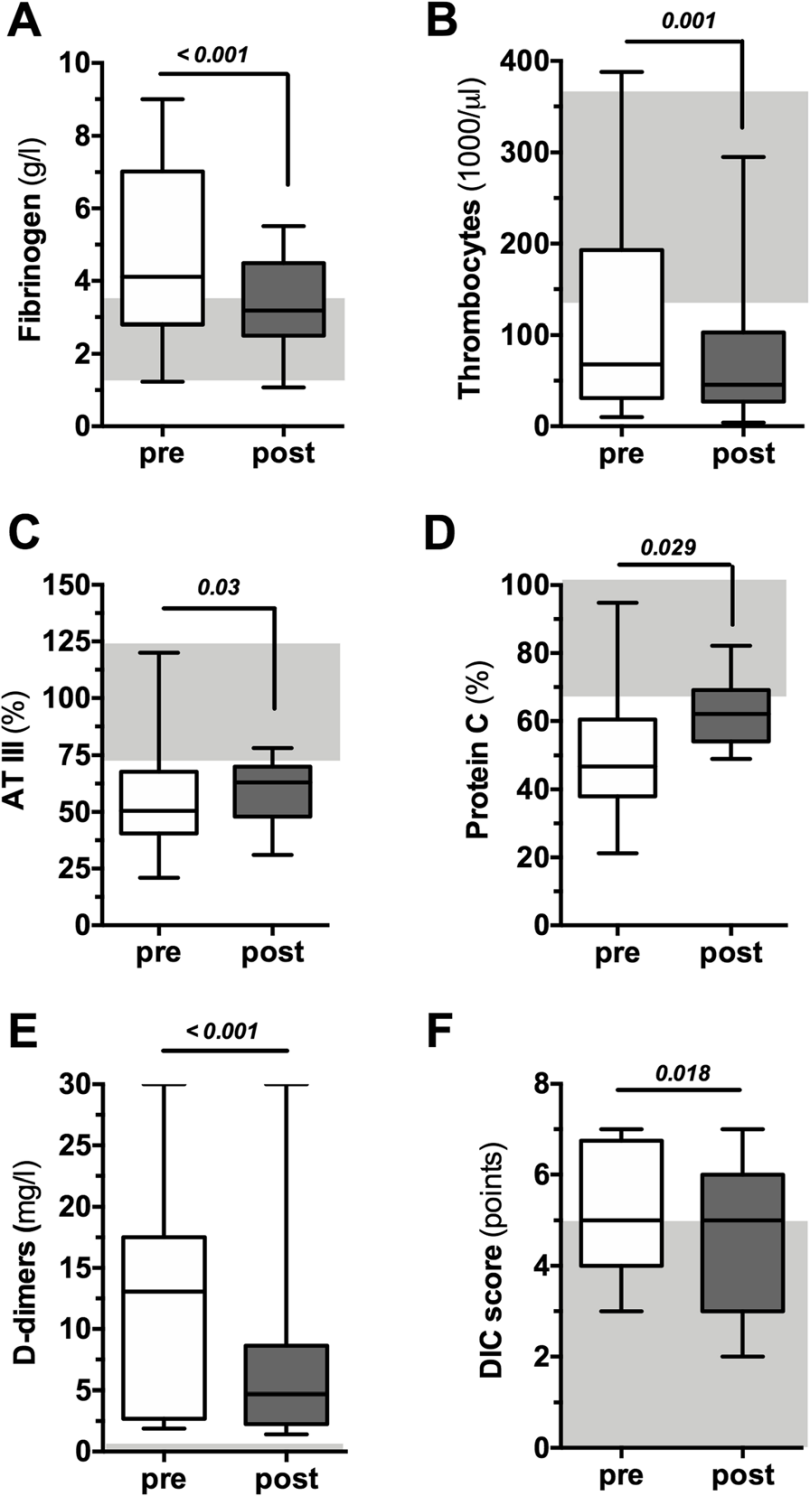
Effect of TPE on routine coagulation parameters, antithrombin-III, protein C, and D-dimers. Box and whisker blots showing fibrinogen (**a**), platelet count (**b**), antithrombin-III activity (**c**), protein C activity (**d**), D-dimer concentration (**e**), and DIC score (**f**) immediately before and post (=after) TPE. Gray areas highlight the reference range for healthy individuals

Fibrinogen was elevated before and slightly decreased after TPE to values considered in the normal range (4.1 (2.8–7) g/L vs. 3.2 (2.5–4.5) g/L, *p* < 0.001, Fig. 2a). As an indicator of sepsis-associated coagulopathy, we observed a median SIC score at inclusion of 5 (4–6) and low platelet counts before TPE. Of note, platelets were even lower (68 (31–193) vs. 46 (27–103), *p* = 0.001, Fig. 2b) after TPE.

Both antithrombotic acting proteins ATIII and protein C activity were markedly reduced before and significantly raised following TPE (ATIII, 51 (41–61) % vs. 63 (48–70) %, *p* = 0.029, Fig. 2c; protein C, 47 (38–60) % vs. 62 (54–69) %, *p* = 0.029, Fig. 2d). D-dimers were severely elevated in all patients and significantly declined following TPE (13.1 (2.7–17.5) mg/L vs. 4.7 (2.3–8.6) mg/L, *p* < 0.001, Fig. 2e). In a subset of 20 patients, the DIC score could be determined. The DIC score significantly decreased following TPE (5.1 ± 1.5 points vs. 4.5 ± 1.5 points, *p* = 0.018, Fig. 2f).

### Effect of TPE on ADAMTS13, vWF:Ag, and vWF:Ag/ADAMTS-13 ratio

**Fig. 3:**
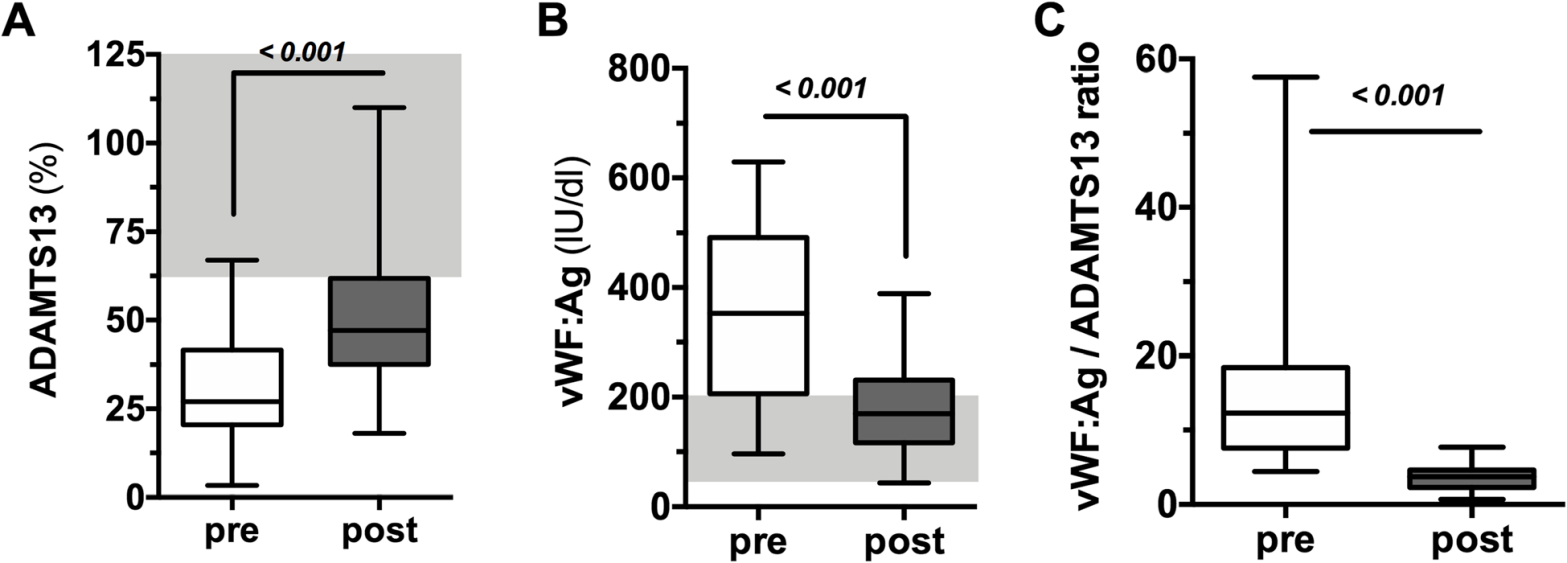
Effect of TPE on ADAMTS13, vWF:Ag, and vWF:Ag/ADAMTS-13 ratio. Box and whisker blots showing ADAMTS13 activity (**a**), vWF:Ag (**b**), and vWF:Ag/ADAMTS13 ratio (**c**) immediately before start and post (=after) TPE. Gray areas highlight the reference range for healthy individuals

ADAMTS13 was reduced below normal limits of 50% before TPE in 29/31 (94%) patients. Moreover, a subset of 17/31 (55%) patients showed severely reduced ADAMTS13 activity below 30%. ADAMTS13 increased from 27 (21–42) % before to 47 (38–62) % after TPE (*p* < 0.001, Fig. 3a).

In contrast, vWF:Ag was increased at study inclusion and reduced by TPE (353 (206–492) IU/dL vs. 170 (117–232) IU/dL, *p* < 0.001, Fig. 3b).

Consequentially, the elevated ratio of vWF:Ag to ADAMTS13 at inclusion was significantly reduced by TPE (12.3 (7.6–18.5) vs. 3.7 (2.3–4.6), *p* < 0.001, Fig. 3c).

ULWF were detectable in a significant amount before TPE in 7/31 (23%) patients and were improved in 5 (71%) and completely normalized in 2 of them after TPE (data not shown).

### ADAMTS13 and correlation with clinical parameters of disease severity at inclusion

**Fig. 4:**
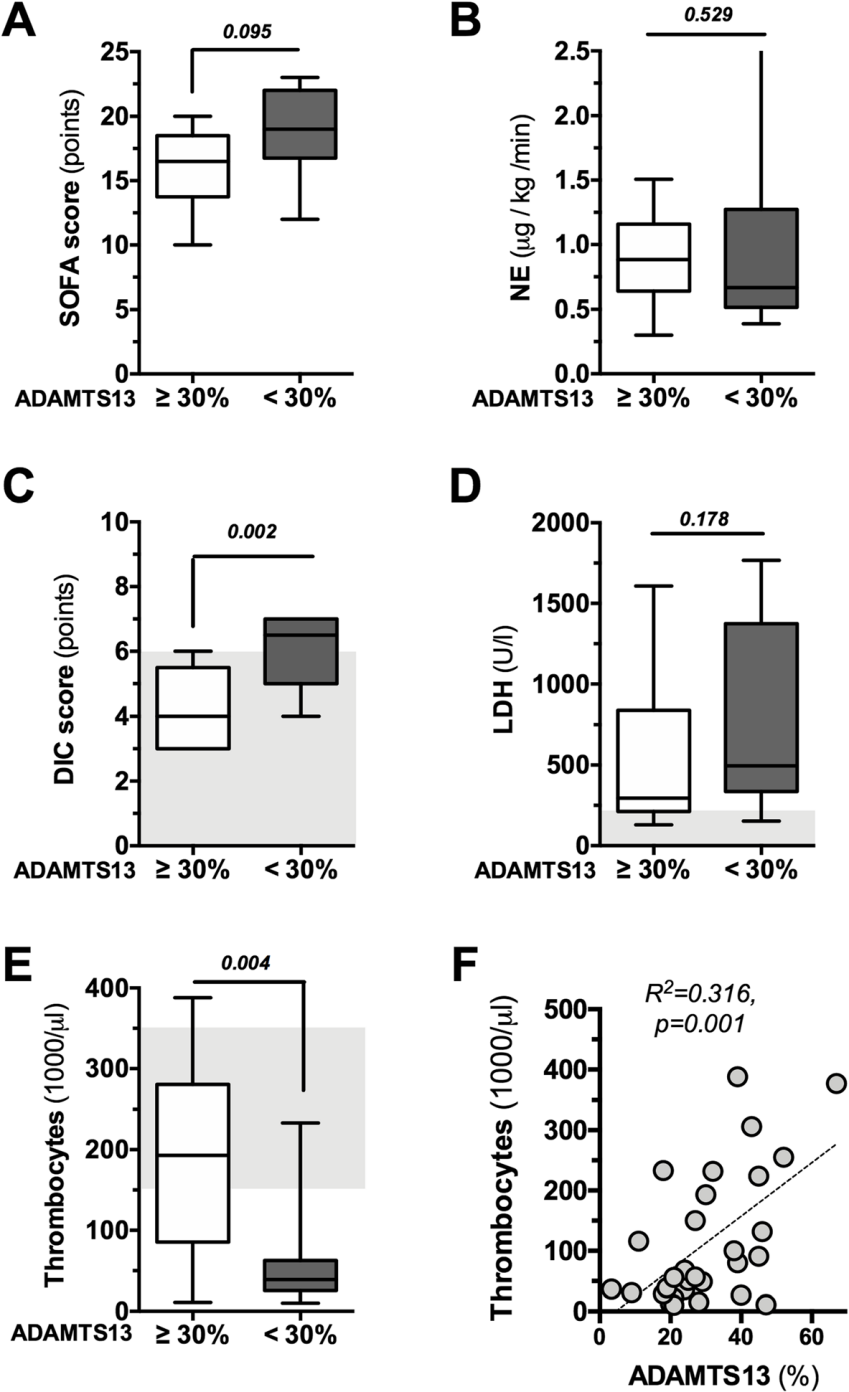
Correlation of ADAMTS13 with clinical parameters of disease severity. Box and whisker blots demonstrating SOFA scores (**a**), NE dose (**b**), DIC score (**c**), LDH (**d**), and platelet count (**e**) for both patients with profound reduction of ADAMTS13 activity (< 30%) and patients with less severe suppression of ADAMTS13 activity (≥ 30%) at the time of inclusion before TPE treatment. **f** Non-linear regression of ADAMTS13 activity to platelet count

Patients with a profound reduction of ADAMTS13 (defined by an activity < 30%) were compared to patients with less severe suppression of ADAMTS13 activity (≥ 30%) in terms of clinical parameters at the time of inclusion before TPE treatment. Patients with ADAMTS13 activity ≥ 30% showed a trend to less organ dysfunction as indicated by lower SOFA scores (16 (14–19) vs. 19 (17–22), *p* = 0.095, Fig. 4a) and comparable severity of hemodynamic shock (NE dose, 0.884 (0.639–1.158) μg/kg/min vs. 0.667 (0.516–1.273) μg/kg/min, *p* = 0.529, Fig. 4b). However, the median DIC score was higher in patients with ADAMTS13 activity < 30% (6.5 (5–7) points vs. 4 (3–5.5) points, *p* = 0.002, Fig. 4c).

Median LDH was 496 (336–1375) U/L in patients with ADAMTS13 < 30%, compared to 295 (212–839) U/L if the activity was ≥ 30% (*p* = 0.178, Fig. 4d). Platelets were severely reduced in patients with ADAMTS13 < 30% (39 (26–63) × 1000/μL and only slightly reduced in those with ADAMTS13 ≥ 30% (193 (86–281) × 1000/μL, *p* = 0.004, Fig. 4e). Non-linear regression yielded a significant correlation between ADAMTS13 activity and platelet count (*p* = 0.001, *R*^2^ = 0.316, Fig. 4f).

### ADAMTS13, vWF:Ag, and survival

The overall 30-day survival was 42% (13/31 patients), and the mean time from termination of TPE treatment to death was 8.4 ± 8.2 days. Survival was 54% (7/13) in patients with ADAMTS13 ≥ 30% and 35% (6/17) in patients with ADAMTS13 < 30% measured at inclusion before performing TPE treatment. The hazard ratio (HR) was 1.46 (95% CI 0.56–3.79, *p* = 0.45, Suppl. Figure 1). ADAMTS13 and vWF:Ag were comparable between surviving and non-surviving patients, both at inclusion and after TPE treatment (Suppl. Figure 2A and C). ADAMTS13 activity was raised by TPE by 82 (50–117) % in survivors and by 72 (16–126) % in non-survivors (*p* = 0.589, Suppl. Figure 2B). Contrastively, vWF:Ag was reduced by TPE by 54% (41–64) in surviving patients and by 47% (37–67) in non-surviving patients (*p* = 0.923, Suppl. Figure 2D).

In terms of shock reversal, we defined subgroups that demonstrated (1) any reduction of NE and (2) a more marked NE reduction by more than 20% of baseline NE dose following TPE treatment (Suppl. Figure 2E and F). TPE-responsive patients with regard to hemodynamic stabilization showed an increase of ADAMTS13 by 83 (35–128) % while patients without any NE dose reduction by 60% (20–104) (*p* = 0.482, Suppl. Figure 2E). ADAMTS13 activity changed by 86 (38–128) % in patients with a NE dose reduction of greater than 20% and by 60% (27–146) in patients with NE dose reduction of ≤ 20% (*p* = 0.513, Suppl. Figure 2F).

## Discussion

This prospective single-center explorative study examined the effects of early TPE on endothelial activation and coagulation-related parameters in septic shock. Patients included in this investigation experienced a severe form of septic shock as indicated by exceedingly high NE requirement, inflammatory markers, and a high prevalence of multi-organ failure. Low platelet counts with high DIC scores indicated the possibility of overt DIC in these patients. However, traditional DIC scores like the ISTH-DIC Score have been reported to have a poor sensitivity, especially in infectious diseases [29]. The SIC score has been suggested recently to be more specific in sepsis [26] but has been questioned in a validation cohort, as the ISTH score had greater power than the SIC score in predicting ICU mortality in septic patients [30]. We acknowledge the limitation of clinical scores to adequately comprehend DIC in septic patients. In this report, we have followed the current expert opinions that recommend to use the SIC score as a screening tool to identify patients with possible sepsis-associated coagulopathy and subsequentially calculate an overt DIC score like the ISTH score [29]. At the same time, hyper-fibrinolysis as indicated by elevated D-dimer concentrations was present. Patients with septic shock displayed a significant shift of the hemostatic balance, characterized by activation of pro-coagulant proteins and attenuation of anti-coagulant activity at the same time. In this sense, pro-coagulatory factors such as vWF-Ag, ULVWF, and fibrinogen were markedly increased at inclusion while anti-coagulant proteins such as ATIII, protein C, and ADAMTS13 were depleted. These observations are well in line with two previous investigations describing these changes in septic patients [14, 20]. Especially, the vWF-cleaving protease ADAMTS13 was reduced in 94% and severely reduced in 55% of patients. This deficiency of ADAMTS13 was associated with both high vWF:Ag and ULVWF concentrations as well as with thrombocytopenia and increased LDH concentrations. Both changes are characteristic for the presence of thrombotic microangiopathy (TMA) with ongoing hemolysis as a consequence of low ADAMTS13 activity. These cardinal features of TMA by a relative deficiency in ADAMTS13 activity were described previously in septic patients and were associated with worse outcomes [13, 17, 19].

In this analysis, we observed higher DIC scores in patients with low ADAMTS13. In contrast, an earlier study by Peigne et al. found no correlation between the DIC score and ADAMTS13 [18]. A possible explanation concerning these contradictory results might be the differences in the severity and duration of septic shock as well as the severity of organ failures between these two cohorts. Peigne et al. described the lower need for both mechanical ventilation and renal replacement therapy. Additionally, inflammatory activity indicated by IL-6 concentration was more than ten times higher in our cohort with a median (IQR) IL-6 of 10,810 pg/mL (2538–27,570) compared to 895 pg/mL (330–1843) in the study by Peigne et al.

The clinical relevance of reduced ADAMTS13 is well known from a disease termed TTP in which the formation of ULVWF multimers due to reduced ADAMTS13 leads to thrombocytopenia, hemolysis, AKI, and (often severe) neurological symptoms. Supplementation of ADAMTS13—that is found in high concentration in the plasma from healthy donors—by TPE can (in some cases within minutes) positively affect these symptoms.

The question arises if it is possible to influence the slightly more complex dysbalance of the hemostatic system in clinical sepsis scenario. Treatment with activated protein C (APC) has demonstrated efficacy in selected patients, e.g., with purpura fulminans [7, 8], but has failed in larger more general sepsis populations [10]. No recombinant ADAMTS13 is available until now, and regular renal replacement regimens have no ability to reduce pro-coagulant factors. We hypothesized that the exchange of septic shock plasma with fresh frozen plasma (FFP) from healthy donors does not only lead to a reduction of harmful proteins but also rather replenishes protective factors that had been consumed by sepsis analogously to TTP. In fact, a key finding of our analysis is that TPE might have the potential to partially attenuate this dysbalance by removing pro- and simultaneously replacing anti-coagulant factors.

A high vWF:Ag/ADAMTS13 ratio has been associated previously with the development of TMA, higher severity of shock, degree of organ failure, and worse outcomes in patients with septic shock [20]. Here, we not only can confirm the elevation of this ratio in septic shock but we can also demonstrate a putative therapeutic strategy to recalibrate this disequilibrium in the coagulation process.

This study has important limitations, mainly its small sample size affecting the power of the results, the single-center setting, and the lack of a clinical correlate that reflects the actual microcirculatory state. Currently available methods in clinical use for microvascular perfusion monitoring such as biomarkers of endothelial damage/activation biomarkers), videomicroscopic techniques, tissue oxygenation evaluation techniques, and partial pressure of carbon dioxide (pCO_2_)-based evaluation techniques were not employed in this study. Unfortunately, the size of this study was too small to demonstrate significant differences in ADAMTS13, vWF:Ag, or the presence of ULVWF between survivors and non-survivors or patients with and without shock reversal by TPE, respectively. As this study was a single-center study, there is a potential for patient selection bias. In addition, the intervention was administered at a fixed dose, which precludes us from providing data on the effects at different dosages or time frames.

## Conclusions

In this prospective study, septic shock was associated with activation of pro-coagulant pathways such as vWF:Ag and ultra-large VWF multimers and consumption of anti-coagulant factors such as ATIII, protein C, and ADAMTS13 activities. Early TPE treatment partially attenuated this dysbalance by removing pro- and replacing anti-coagulant factors. It has yet to be determined by an appropriately powered RCT if TPE might improve the outcome in septic patients.

## Supplementary information

**Additional file 1: Figure S1.** Thirty-day survival. Kaplan Meier graphs showing the 30-day survival course in (A) the overall cohort (41.9% survival (13/31 patients)) as well as (B) in patients with ADAMTS13 ≥ 30% (53.9% survival (7/13)) and (C) in patients with ADAMTS13 < 30% (35.3% survival (6/17)). ADAMTS13 activity was measured at inclusion before performing TPE treatment.

**Additional file 2: Figure S2.** ADAMTS13 and vWF:Ag and clinical outcome. Box and whisker blots demonstrating ADAMTS13 activity (A) and vWF:Ag (C) pre- and post TPE as well as % change of ADAMTS13 activity (B) and vWF:Ag (D) by TPE. Percentage change of ADAMTS13 activity is also displayed dependent on achieved reduction of NE (E) dose and reduction of NE dose by > 20% (F) following TPE.

## Data Availability

The datasets used and analyzed during the current study are available from the corresponding author on reasonable request.

## Abbreviations

ADAMTS13: A disintegrin and metalloprotease with a thrombospondin type 1 motif, member 13
AKI: Acute kidney injury
BMI: Body mass index
CRP: C-reactive protein
FFP: Fresh frozen plasma
MAP: Mean arterial pressure
NE: Norepinephrine
PCT: Procalcitonin
RCT: Randomized controlled trial
RRT: Renal replacement therapy
SOFA: Sequential Organ Failure Assessment
TPE: Therapeutic plasma exchange
ULVWF: Ultra-large von Willebrand factor multimers
vWF: von Willebrand factor
vWF:Ag: von Willebrand factor antigen
