# Supplementary figures and images for: Effect of therapeutic plasma exchange on endothelial activation and coagulation-related parameters in septic shock
Source: Crit Care. 2020 Mar 2;24:71. doi: 10.1186/s13054-020-2799-5 (PMC7053051; doi:10.1186/s13054-020-2799-5)

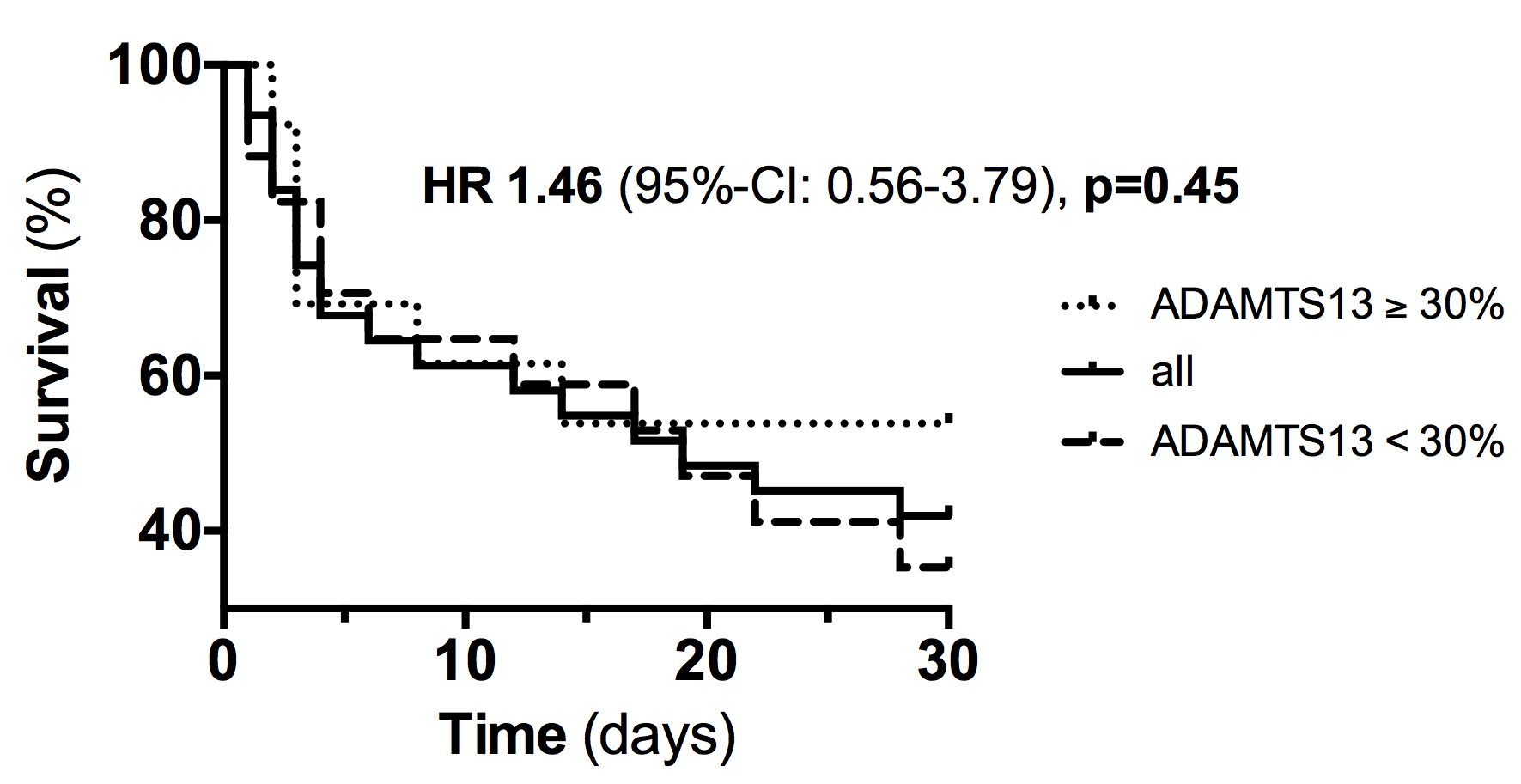

Supplement: Supplementary file 1 — Additional file 1: Figure S1. Thirty-day survival. Kaplan Meier graphs showing the 30-day survival course in (A) the overall cohort (41.9% survival (13/31 patients)) as well as (B) in patients with ADAMTS13 ≥ 30% (53.9% survival (7/13)) and (C) in patients with ADAMTS13 < 30% (35.3% survival (6/17)). ADAMTS13 activity was measured at inclusion before performing TPE treatment. [file 13054_2020_2799_MOESM1_ESM.tiff]

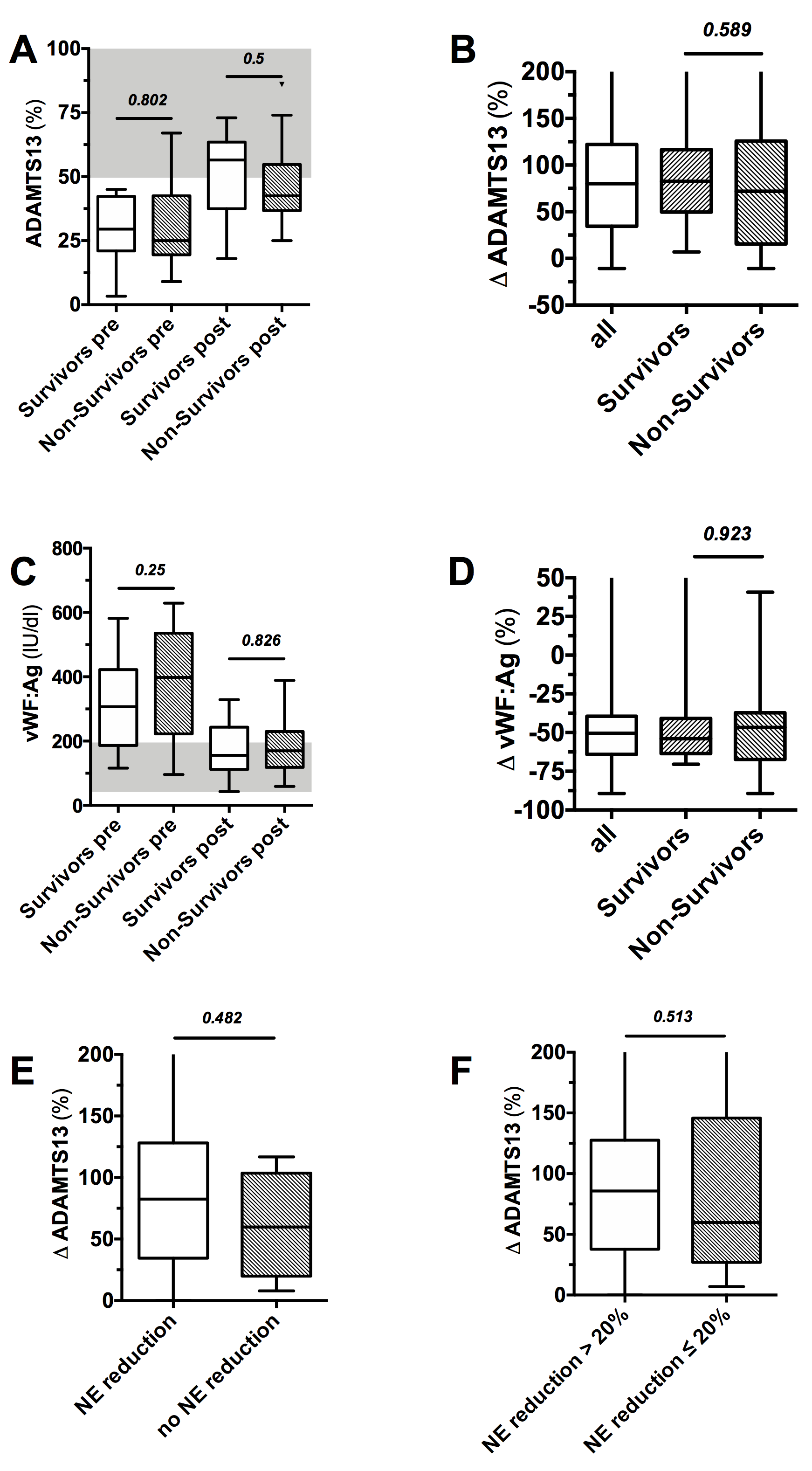

Supplement: Supplementary file 2 — Additional file 2: Figure S2. ADAMTS13 and vWF:Ag and clinical outcome. Box and whisker blots demonstrating ADAMTS13 activity (A) and vWF:Ag (C) pre- and post TPE as well as % change of ADAMTS13 activity (B) and vWF:Ag (D) by TPE. Percentage change of ADAMTS13 activity is also displayed dependent on achieved reduction of NE (E) dose and reduction of NE dose by > 20% (F) following TPE. [file 13054_2020_2799_MOESM2_ESM.tiff]
